# Characterization of a novel polyclonal anti-hypusine antibody

**DOI:** 10.1186/2193-1801-2-421

**Published:** 2013-08-29

**Authors:** Yurika Nishiki, Thomas B Farb, Jessica Friedrich, Krister Bokvist, Raghavendra G Mirmira, Bernhard Maier

**Affiliations:** Department of Pediatrics and the Herman B Wells Center for Pediatric Research, Indiana, University School of Medicine, Indianapolis, IN 46202 USA; Lilly Research Labs, Eli Lilly & Co, Indianapolis, IN 46285 USA; Departments of Medicine, Cellular and Integrative Physiology, and Biochemistry and Molecular Biology, Indiana University School of Medicine, Indianapolis, IN 46202 USA

**Keywords:** Hypusine, eIF5A, Antibody, Recombinant proteins, Cell lines

## Abstract

The translation factor eIF5A is the only protein known to contain the amino acid hypusine, which is formed posttranslationally. Hypusinated eIF5A is necessary for cellular proliferation and responses to extracellular stressors, and has been proposed as a target for pharmacologic therapy. Here, we provide the first comprehensive characterization of a novel polyclonal antibody (IU-88) that specifically recognizes the hypusinated eIF5A. IU-88 will be useful for the investigation of eIF5A biology and for the development of assays recognizing hypusinated eIF5A.

## Introduction

Eukaryotic translation initiation factor 5A-1 and 5A-2 (eIF5A-1 and eIF5A-2—collectively referred to here as eIF5A) are highly conserved proteins whose varied cellular functions include the binding of and nucleocytoplasmic shuttling of specific mRNAs (Kruse et al. 2000; Maier et al. 2010; Xu and Chen 2001), cellular proliferation (Nishimura et al. 2005), and posttranslational stress responses (Li et al. 2010; Moore et al. 2008; Nishiki et al. 2013). Curiously, eIF5A is the only protein containing the amino acid hypusine, which is formed from a lysine residue in a posttranslational reaction involving the enzymes deoxyhypusine synthase (DHS) and deoxyhypusine hydroxylase (DHH) and the substrate spermidine (Park et al. 2010). In the complete absence of deoxyhypusine synthase mouse embryos die at a very early stage of development (Nishimura et al. 2012; Templin et al. 2011). Inhibition of hypusine formation has been suggested to confer cellular survival in certain stress states, such as infections, carcinogenesis, and obesity (Balabanov et al. 2007; Hauber et al. 2005; Robbins et al. 2010; Schwentke et al. 2012). Therefore, identification of the hypusinated form of eIF5A is paramount in the understanding of the biology of the protein. Nevertheless, identification of hypusinated eIF5A has remained a challenge, requiring tedious methods such as isoelectric focusing or two-dimensional gel electrophoresis of cellular extracts. Although prior studies reported the development of antibodies against hypusinated eIF5A (Bergeron et al. 1998; Cracchiolo et al. 2004), their characterizations were limited and utilities of these reagents were not described in subsequent reports. Here, we present the characterization of a novel anti-hypusine antibody reagent, IU-88. We demonstrate that IU-88 selectively recognizes either the deoxyhypusine or hypusine forms of eIF5A in vitro, and that IU-88 specifically recognizes the hypusinated form of eIF5A in cellular extracts by immunoblots and in whole cells by immunocytochemistry.

## Materials and methods

### Cell culture, transfection and DHS inhibition

Human 293T and rat INS-1(832/13) β cells were cultured as described (Hohmeier et al. 2000). Cells were transiently transfected with plasmids encoding EGFP-eIF5A, EGFP-eIF5A(K50A) and EGFP-DHS constructs using Lipofectamine 2000 (Invitrogen) for 16 hours before cell extraction or immunofluorescence analysis. The DHS inhibitor GC7 (Biosearch Technologies) was prepared and used in cell culture as previously described (Maier et al. 2010).

### Reactions in vitro

For in vitro experiments, eIF5A protein was purified from *E. coli* as a GST fusion, after which the GST tag was proteolytically removed. DHS protein was purified from *E. coli* as an N-terminal His6 fusion. Purified human DHH protein was purchased from OriGene. The hypusination reactions in vitro proceeded as previously published (Wolff et al. 2011).

### Antibodies and immunoblotting

The rabbit polyclonal antibody IU-88 against hypusinated human eIF5A was generated in rabbits using the synthetic hypusine-containing peptide C-Ahx-STSKTG[hypusine]HGHAKV-amide by contract to 21^st^ Century Biochemicals. Monoclonal mouse pan-anti-eIF5A antibody was from BD Biosciences and anti-actin antibody was from MP Biomedicals. Immunoblot analysis was visualized using a LiCor Odyssey fluorescence system following electrophoresis on a 4-20% SDS polyacrylamide gel (Maier et al. 2010). Primary antibodies were diluted 1:1500 (IU-88) and 1:10,000 (anti-pan-eIF5A).

### Fluorescence immunocytochemistry

293T cells were fixed in 4% paraformaldehyde and immunocytochemistry proceeded as previously described (Robbins et al. 2010). Antibody dilutions were 1:150 for IU-88 and 1:1000 for anti-pan-eIF5A. 4',6-diamidino-2-phenylindole (DAPI) staining was used to visualize nuclei. A Zeiss LSM-710 microscope was used to visualize cells at magnification x100.

## Results and discussion

**Figure 1:**
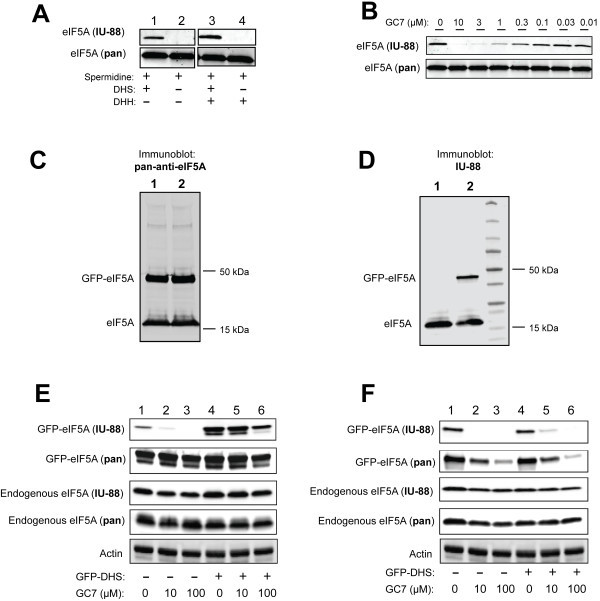
**Immunoblot characterization of polyclonal antibody IU-88.*A****,* Recombinant human eIF5A was treated in vitro with spermidine, DHS, and DHH as indicated, then subjected to polyacrylamide gel electrophoresis and immunoblots analysis using antibody IU-88 or a pan-anti-eIF5A antibody (a BD monoclonal antibody); ***B****,* Recombinant human eIF5A was treated in vitro with spermidine, DHS, and different concentrations of GC7 as indicated, then subjected to polyacrylamide gel electrophoresis and immunoblot analysis using antibody IU-88 or a pan-anti-eIF5A antibody; ***C****,* INS-1 β cells were transfected with a plasmid encoding either GFP-eIF5A(K50A) (*lane 1*) or GFP-eIF5A (*lane* 2), then cell extracts were subjected to polyacrylamide gel electrophoresis and immunoblots analysis using a pan-anti-eIF5A antibody; ***D****,* INS-1 β cells were transfected with a plasmid encoding either GFP-eIF5A(K50A) (*lane 1*) or GFP-eIF5A (*lane* 2), then cell extracts were subjected to polyacrylamide gel electrophoresis and immunoblots analysis using antibody IU-88; ***E****,* 293T cells were transfected with a plasmid encoding GFP-eIF5A, with or without another plasmid encoding DHS (as indicated), and treated with different concentrations of GC7 as indicated. Cell extracts were then subjected to polyacrylamide gel electrophoresis and subsequent immunoblots analysis using antibody IU-88, a pan-anti-eIF5A antibody, and an anti-actin antibody; ***F****,* INS-1 cells were transfected with a plasmid encoding GFP-eIF5A, with or without another plasmid encoding DHS (as indicated), and treated with different concentrations of GC7 as indicated. Cell extracts were then subjected to polyacrylamide gel electrophoresis and subsequent immunoblots analysis using antibody IU-88, a pan-anti-eIF5A antibody, and an anti-actin antibody.

To determine if IU-88 specifically recognizes the deoxyhypusine or hypusine forms of eIF5A, we performed immunoblots of reactions in which recombinant human eIF5A was incubated in vitro with DHS, DHH, spermidine, and/or the potent DHS inhibitor GC7. Figure 1A shows that IU-88 is incapable of recognizing eIF5A when it is incubated with spermidine alone (lane 2) or with DHH+spermidine (lane 4). However, IU-88 recognized eIF5A when co-incubated with DHS+spermidine (lane 1) or DHS+DHH+spermidine (lane 3), suggesting that both the deoxyhypusine and hypusine forms of eIF5A are recognized (although it cannot be determined if both forms are recognized with equal affinity). Increasing the DHS concentration and time of incubation led to increasing eIF5A signal intensity in these studies (data not shown). Co-incubation of the reaction with DHS+spermidine with increasing concentrations of the DHS inhibitor GC7 (up to 10 μM) caused near-complete inhibition of eIF5A signal intensity (Figure 1B). Notably, the differences in eIF5A intensity in these studies were not because of differences in protein loading, since a pan-anti-eIF5A monoclonal antibody (BD) demonstrated equal loading (Figure 1A and B).

Next, we tested the ability of IU-88 to specifically recognize hypusinated eIF5A in whole cellular extract by immunoblotting. As shown in the full gel image in Figure 1C, when extracts from rat-derived INS-1 islet β cells are used in immunoblotting, IU-88 recognizes only a single protein species at ~17 kDa, corresponding to the known molecular weight of eIF5A. Transfection of a plasmid encoding either a human EGFP-eIF5A(K50A) fusion protein (which is not capable of being hypusinated) or a human EGFP-eIF5A fusion protein results in the appearance of a protein species at ~44 kDa only with the EGFP-eIF5A transfection (Figure 1C, compare lanes 1 and 2). When these transfections are immunoblotted using an antibody against GFP, both EGFP-eIF5A(K50A) and EGFP-eIF5A are recognized (Figure 1D). These data demonstrate specificity of IU-88 in recognizing only eIF5A in total cellular protein, and also suggest that IU-88 only recognizes transfected eIF5A proteins that have the capability to be hypusinated. To investigate in greater detail the utility of IU-88 to distinguish hypusination in cellular extracts, we performed additional studies in human-derived 293T cells and rat-derived INS-1 cells. As shown in Figure 1E, when human 293T cells are transfected with GFP-eIF5A, a weak but detectable signal corresponding to GFP-eIF5A is observed using IU-88 (lane 1). This signal decreases further upon co-incubation with increasing concentrations of GC7 (Figure 1E, lanes 2 and 3), suggesting that IU-88 is recognizing the hypusine-specific form. Interestingly, when exogenous DHS is introduced by co-transfection of a GFP-DHS fusion protein-encoding vector, there is a dramatic increase in GFP-eIF5A signal as detected by IU-88 (Figure 1E, lane 4) with corresponding decrease in the presence of GC7 (lanes 5 and 6), suggesting that DHS protein levels may be limiting in the ability of 293T cells to hypusinate eIF5A—a finding that is also observed in human-derived HeLa cells (Lee et al. 2009). INS-1 β cells, by contrast, reveal a significantly different picture. As shown in Figure 1F, transfection of a plasmid encoding GFP-DHS did not enhance the signal observed with either GFP-eIF5A or endogenous eIF5A, suggesting that DHS is not limiting in the ability of INS-1 cells to hypusinate eIF5A. Interestingly, whereas increasing GC7 concentrations reduce the GFP-eIF5A signal observed with IU-88, it also reduces the signal observed with the pan-anti-eIF5A antibody (Figure 1F). This result suggests that INS-1 β cells may be unique in their requirement for hypusination to maintain production of eIF5A itself.

Figure 1E and F show that GC7 incubation also reduces, but only slightly, the level of endogenous eIF5A, as detected by IU-88 (and perhaps more-so in INS-1 cells than in 293T cells). Because IU-88 measures only steady-state levels of hypusinated eIF5A (as opposed to rate of hypusine formation—as measured by 3H-spermidine uptake studies, ref. (Maier et al. 2010)), this observation may reflect the long half-life (6–24 h) of the hypusinated eIF5A protein in mammalian cells (Gerner et al. 1986; Maier et al. 2010). More effective depletion of the hypusinated eIF5A species with GC7 may require longer periods of incubation.

**Figure 2:**
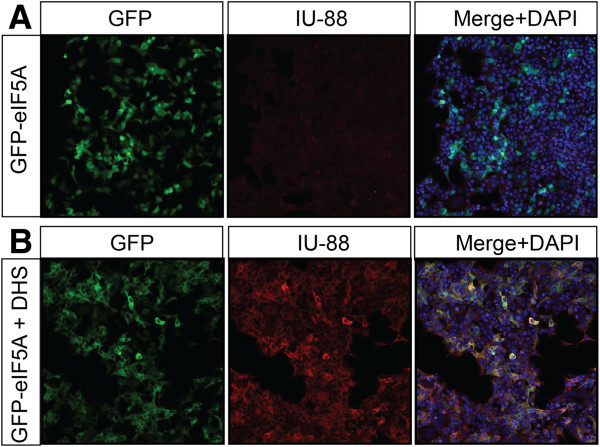
**Immunocytochemistry of 293T cells using antibody IU-88.*A***, 293T cells were transfected with a plasmid encoding GFP-eIF5A, then immunostained using IU-88 and counterstained with DAPI to visualize nuclei; ***B***, 293T cells were transfected with plasmids encoding GFP-eIF5A and DHS, then immunostained using IU-88 and counterstained with DAPI to visualize nuclei. In *panels****A****and****B***, GFP is visualized in the *green* channel, IU-88 is visualized in the *red* channel, and DAPI is visualized in the *blue* channel. Magnification x100.

Because IU-88 shows specificity for hypusinated eIF5A, we next asked whether IU-88 can recognize protein in the context of fluorescence immunocytochemistry. 293T cells were transfected with a plasmid encoding GFP-eIF5A, then stained with DAPI (to visualize nuclei) and immunostained using IU-88. As shown in Figure 2A staining intensity with IU-88 was weak, consistent with the immunoblot in Figure 1D. However, when cells were cotransfected with GFP-DHS, a striking increase in cytoplasmic staining was observed with IU-88 (Figure 2B). A notable observation in Figure 2 is the apparent relocalization of eIF5A from a pan-nuclear/cytoplasmic distribution to a primarily cytoplasmic distribution in the presence of DHS overexpression (c.f. GFP-eIF5A staining in Figure 2A and B). This result suggests that hypusinated eIF5A may occupy primarily a cytoplasmic distribution, as proposed in prior studies (Lee et al. 2009; Maier et al. 2010). However, recent studies have also implicated a role for acetylation in eIF5A compartmentation (Ishfaq et al. 2012), suggesting perhaps a more complex interplay between hypusination and other modifications in the function and subcellular localization of the factor.

Taken together, our results verify the specificity and utility of IU-88 in detecting a specifically modified form of eIF5A. Depending upon the application, an important caveat to the use of IU-88 is its inability to distinguish between the deoxyhypusinated and hypusinated forms of eIF5A. Although the relative significance of the deoxyhypusinated vs. hypusinated forms of eIF5A remains unclear, the low substrate Km of DHH relative to DHS means that the majority of eIF5A in cells is likely present in the fully hypusinated form (Park et al. 2010). Nevertheless, IU-88 represents an especially useful reagent for the assessment of at least the activity of DHS in cells. Also, because most pharmacologic approaches to inhibiting the hypusination reaction have focused on inhibition of the higher Km enzyme DHS, IU-88 would also serve as an important reagent for assessing DHS activity in drug screening studies.

## Availability and requirements

None.
